# Midwifery

**Published:** 1837-04

**Authors:** 


### MIDWIFERY.

*Two Cases of complete Recovery from Laceration of the Posterior Part of the Vagina and Cervix Uteri.* By J. TOOGOOD, Esq., Surgeon to the Bridgewater Infirmary.

THESE cases are interesting, from the rareness of recovery after such an accident. They are related with commendable brevity. In the first case there was "very extensive laceration of the posterior part of the vagina, which extended to the cervix uteri:" both Mr. T. and another surgeon, believing the case hopeless, "passed the hand freely through a large rent into the cavity of the abdomen, and felt the different viscera distinctly, and the abdominal aorta." Although given up, this woman gradually and rapidly recovered. Mr. T. is disposed to attribute the recovery to the great attention paid to the case, and to the *omission* of bloodletting on the accession of reaction with abdominal pain, &c. He was so satisfied that the loss of even a small quantity of blood, although attended perhaps with immediate relief, would in all probability have been fatal, that he "determined to trust to opium, cordials, nourishment, and perfect quiet;" and the result seemed to justify the treatment.

In the second case, there was also "a considerable laceration of the posterior part of the vagina, in which the cervix uteri was involved, and the intestines were lying in the vagina." The treatment is not given in detail, but it would appear that large opiates were the early remedies depended on. This patient also completely recovered. *British Annals of Medicine, Jan.* 20, 1837.

